# The prevalence of occupational injury and its associated factors in Ethiopia: a systematic review and meta-analysis

**DOI:** 10.1186/s12995-020-00265-0

**Published:** 2020-06-03

**Authors:** Yoseph Merkeb Alamneh, Abriham Zegeye Wondifraw, Ayenew Negesse, Daniel Bekele Ketema, Tadesse Yirga Akalu

**Affiliations:** 1 Department of Biomedical Sciences, School of Medicine, Debre Markos University, P.O. Box 269, Debre Markos, Ethiopia; 2 Department of Human Nutrition and Food Sciences, College of Health Sciences, Debre Markos University, P.O. Box 269, Debre Markos, Ethiopia; 3 Academic Center of Excellence for Human Nutrition, School of Human Nutrition, Food Science and Technology, Hawassa University, Awasa, Ethiopia; 4 Department of Public Health, College of Health Sciences, Debre Markos University, P.O. Box 269, Debre Markos, Ethiopia; 5 Department of Nursing, College of Health Sciences, Debre Markos University, P.O. Box 269, Debre Markos, Ethiopia

**Keywords:** Occupational injury, Pooled prevalence, Ethiopia, Systematic review, Meta-analysis

## Abstract

**Background:**

Occupation related fatality and mortality rate is becoming the devastating issue globally as reported by the International Labor Organization (ILO). Though there are reports about exposure and burden of occupational injury from the regional states, the studies were fragmented and inconclusive ones at the national level. Hence, the authors’ intention being to come up with the national pooled estimates of occupation related injury and the associated factors in Ethiopia.

**Methods:**

The international reputable databases (PubMed, Google Scholar, ScienceDirect and Cochrane Library), cross-referencing and manual search strategies were explored rigorously following Preferred Reporting Items for Systematic Reviews and Meta-Analysis Protocol (PRISMA-P) guideline. Studies that reported the prevalence of occupational injury were included for this systematic review and meta-analysis. The Newcastle-Ottawa quality assessment tool scale for cross-sectional studies was used for the critical appraisal of the studies. The heterogeneity between the studies was checked using Cochran Q statistic with the inverse variance (I^2^) value. Random effects meta-analysis was considered assess the summative effect size of occupational injury and the factors associated with it. Subgroup analysis and meta-regression were also employed to identify the possible source of heterogeneity and factors associated with occupational injury respectively. Both Egger’s and Begg’s test with the *p*-value less than 5% were used to declare the presence of publication bias.

**Results:**

A total of 23 original studies were considered to estimate the pooled effect size of occupational injury in Ethiopia. The pooled prevalence of occupational injury in Ethiopia was 44.66% (95% CI: 43.83, 45.49). Based on the subgroup analysis, the highest prevalence of occupational injury was reported from the construction sites (50.8%) in particular of the Addis Ababa city administration (49.5.Being male workers [OR = 1.46 (95% CI: 1.01, 2.11)], working more than eight hours per day [OR = 2.84 (95% CI: 1.81, 4.46)], absence of supervision for labor workers [OR = 1.60 (95% CI: 1.08, 2.37)], lack of personal protective equipment [OR = 3.01 (95% CI: 1.61, 5.63)] and lack of occupational health and safety training [OR = 1.49 (95% CI: 1.15, 1.92)] had increased odds of occupational injury.

**Conclusion:**

Based on this systematic review and meta-analysis, it is concluded that nearly half of the labor workers in Ethiopia were experienced occupational injury. This issue was more encountered among the labor workers of construction sites and whose working place were at the Addis Ababa city administration respectively. Being male sex, working more than eight hours per day, lack of personal protective equipment, lack of supervision, and lack of training about occupational health and safety had increased odds of occupational injury in Ethiopia. Hence, the concerned body should give special emphasis for all the explored factors in order to minimize occupation related injury, mortality and morbidity in the country.

## Background

Occupational injury is any physical injury that affects a labor worker while working [1]. Occupational accidents, work injury, work-related injury, work accidents, and work-related accidents are synonymous phrases for occupational injury [2]. Around 20 ILO conventions have been ratified by Ethiopia in particular of the Occupational Safety and Health Convention 1981 (No. 155). According to the Labor Proclamation No.377/2003 form the Ministry of Labor and Social Affairs in Ethiopia, the government itself is responsible for supervising the labor administration, working conditions, occupational safety and health [3]. Though there is favorable policy and regulatory frameworks in Ethiopia, its monitoring systems and laboratory investigations are inadequate. Even the internal infrastructural capability is also weak and it can’t help to identify and determine workplace injury too. Globally, occupational injury is becoming a public health emergency. It killed more than 300,000 labor force every year and it caused many more cases of disability [4]. Health at work and healthy work environments are an input to the national economies via improved productivity, product quality, work motivation, job satisfaction and overall quality of the worker’s life and society [5]. Though occupational injury is preventable, it is becoming amongst the major public health problems that causes an estimated economic loss of 5–10% growth national product beyond its increased risk of fatality and morbidity rates [6–8], in which 14 death reported per 100,000 workers [9, 10]. This issue is more pronounced in the Sub-Saharan Africa too [11]. Currently, constructions, human health and social work activities, Sewerage, waste management and remediation activities, and manufacturing industries are increasing in Ethiopia. Consequently, the problem of occupational injury is increasing due to lack of safe working conditions. In Ethiopia, reports indicated that only 5 to 10% of workers have access to occupational health services in their respective workplaces. As mentioned earlier, occupational injury has direct economic costs, have a wide range of social consequences including both psychological and behavioral responses for the worker, family and for the community at all. Beyond its chronic consequences of disability, it has also tremendous impact on economy at individual, household and national level. This is because the disability itself kept people out of work longer than any other disabled condition; which is in-turn its cost is easily calculated via missed working days, related costs of replacing jobs, the cost of disability itself, the cost needed to give medical care, and the substitute labor [12].

In Ethiopia, different fragmented and few studies have been conducted to assess the prevalence of occupational injury and associated factors. Employment pattern, drinking alcohol, sleep disorder, job satisfaction, use of personal protective equipment, working hours per week, health and safety training, work experience, work schedule, daily supervision, smoking, pollution and occupational safety are expected to be the possible factors for the occurrence of occupational injury [9]. However, the pocket studies were fragmented and did not representative of the national estimates. Therefore, the main objective of this systematic review and meta-analysis was to estimate the national pooled prevalence and associated factors of occupational injury. These findings will be an input for policymakers and program planners of occupational health and safety in order to inform, plan, implement and evaluate health promotion policies and strategies which are crucial for preventing occupational injury.

## Method and materials

### Study design and setting

This systematic review and meta-analysis was aimed at assessing the prevalence and associated factors of occupational injury in Ethiopia. This country is located in the horn of Africa. It is bounded by Eritrea to the north, Djibouti, and Somalia to the east, Sudan and South Sudan to the west, and Kenya to the south [13]. According to the World Bank compilation of development indicators, the total labor force in Ethiopia was estimated as 53, 746, 763 (22.11% of total employment) in 2019; of which approximately 1% of the total workforce were engaged in manufacturing and construction industry. Moreover, Ethiopia is one of the countries in the world with a low health workforce density of 0.7/1000 population. This is below World Health Organization (WHO) recommendation in which the minimum threshold level is 2.3 health workers per 1000 population for better accessibility of essential services and to decrease the risk of work related injury as a result of work overload [14, 15].

### Searching strategies

We reviewed different kinds of literature such as both Published and unpublished studies based on the eligibility criteria of this study. The Preferred Reporting Items for Systematic Reviews and Meta-Analysis Protocol (PRISMA- P) [16] guideline was used to confirm the scientific accuracy. PubMed, Google Scholar, ScienceDirect and Cochrane Library databases were explored. The key terms used in building the search strategy for the Databases were prevalence, magnitude, occupational injury, occupational accidents, work-related injury, work-related accidents, and Ethiopia. The key terms were combined using Boolean operators to search the electronic databases. In addition, all fields and mesh terms were used whilst for the advanced PubMed searching.

### Eligibility criteria

#### Inclusion criteria

This systematic review and meta-analysis considered all studies which were conducted in Ethiopia. Studies which reported both the prevalence and associated factors were included regardless of their publication, publication time and study designs.

#### Exclusion criteria

Based on the eligibility criteria, we read their titles and abstracts. If studies were relevant for our review, we examined the full texts. Those papers not fully accessed at the time of our search process were excluded after contact was attempted with the principal investigator through email at least two times. The reason for the exclusion of these articles is that we were unable to assess the quality of each article in the absence of their full texts. Moreover, studies which did not report our outcome of interest were excluded after reviewing their full texts. Once more, studies with poor quality as pre-settled criteria and review articles were also excluded from this systematic review and meta-analysis.

#### Quality assessment

The Database search results were combined and duplicate articles were removed manually using Endnote (version X8.2). The Newcastle-Ottawa quality assessment tool scale for cross-sectional studies was used to assess the quality of each study [17]. The modified Newcastle-Ottawa scales consists of three sections. The first section tool is rated from five stars for methodological evaluation. The second section tool is ranked from three stars for comparability assessment. The third section tool is evaluated from two points that deals with the statistical analysis and the outcome of each study. Three independent reviewers critically appraised each individual paper. Disagreements between those reviewers were solved by discussion. If not, a third reviewer was involved to resolve inconsistencies among the three independent reviewers. The original studies which scored ≥6 out of 10 were considered as high quality and included for the final meta-analysis.

### Data extraction

Data were extracted by three authors via a standardized data extraction spreadsheet. The data extraction spreadsheet was piloted on 5 randomly selected papers and modified accordingly. Data extraction sheet included study characteristics such as: (1) Authors’ name, year, region, study or publication year, study design, study setting and actual sample size; (2) prevalence of occupational injury (3) information on gender distribution, supervision, working time, training about occupational health and safety, and about protective equipment from work-related hazard.

### Outcome of measurement

This study had one main outcome. It was the prevalence of occupational injury. It was calculated as the number of individuals who experienced occupational injury divided by a total number of individuals who were at risk of developing occupational injury and multiplied by 100%.

### Data analysis

The extracted data were entered into the computer using the excel sheet and imported to STATA 14 for analysis. Evidence of publication bias and heterogeneity was assessed. Both Egger’s and Begg’s test were used with a *p*-value of less than 0.05 as a cutoff point to declare the presence of publication bias [18]. Heterogeneity across studies was checked using Cochran Q statistic with the inverse variance (I^2^) of 30 to 60%,50 to 90%, and 75 to 100% with moderate, substantial and considerable heterogeneity across individual studies [19]. We used the forest plot to visualize the presence of heterogeneity. A *p*-value of less than 0.05 was also used to declare the presence of heterogeneity across studies. Potential differences between the studies were explored by subgroup analysis and meta-regression. The finding was presented via forest plot with respective odds ratio and 95% confidence intervals using random effects meta-analysis (DerSimonian and Laird) model. The influence of heterogeneity across studies on the meta-analysis was calculated by I-square statistic (TAU) and a cutoff point of 50% was used to declare substantial heterogeneity.

## Results

### Selection and identification of studies

**Fig. 1:**
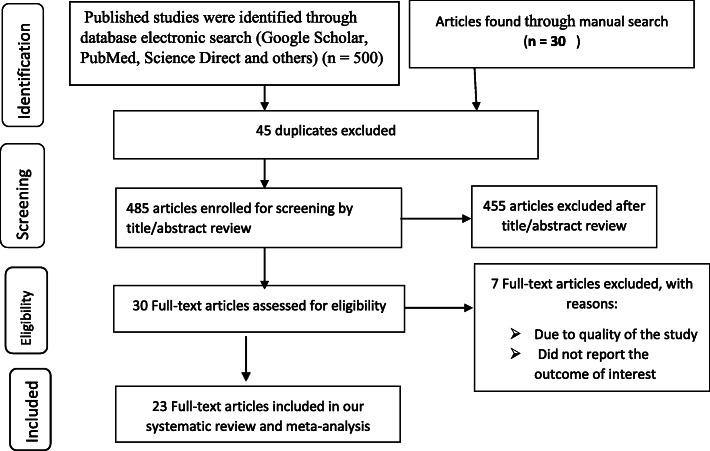
PRISMA flow diagram of included studies to estimate the pooled prevalence and associated factors of occupational injuries in Ethiopia, 2019

From 30 November up to 30 December 2018, a total of 530 studies were identified using electronic searches (500 articles) and manual search (30 articles). Of which, 45 studies were excluded due to duplication. After reviewing their titles and abstracts, 425 studies were excluded as they were irrelevant. The remaining 30 full-text articles were assessed for eligibility. Finally, 23 studies fulfilled the eligibility criteria and included for this systematic review and meta-analysis (Fig. 1).

### Characteristics of included studies

A total of 23 original studies with a total sample of10996 participants were included to estimate the pooled prevalence of occupational injury and its associated factors. Amongst those original studies, five studies were conducted in Addis Ababa [9, 20–23], two studies for each were conducted in Hawassa [24, 25], Arba Minch [26, 27], Gondar [28, 29], Amhara [30, 31], and one study for each was also conducted in Bahir Dar and Gondar [32], Gambella [33], Mekelle [34], Mezan – Aman [35], Afar [36], Kombolcha [37], Bale [38], Jimma [39], Muger Cement manufacturing Industry [2] and Bahir Dar [40] (Table 1).

**Table 1:** Characteristics of the included studies to estimate the prevalence and associated factors of occupational injury in Ethiopia

Authors name	Injury site	Study area	Publication year	Design	Sample size	Response rate	Prevalence (95% CI)	Quality score
Chercos and Berhanu [33]	Manufacturing Industry	Gambella	2017	Cross-sectional	449	100	36.70 (32.24, 41.16)	6
Y. Habtu et al. [9]	Manufacturing Industry	Addis Ababa	2014	Cross-sectional	829	97.99	48.90 (45.50, 52.30)	7
Berhe, et al. [34]	Manufacturing Industry	Mekelle	2015	Cross-sectional	758	97.9	58.20 (54.69, 61.71)	6
Andualem et al. [35]	Manufacturing Industry	Mizan-Aman	2017	Cross-sectional	219	100	45.20 (38.60, 51.79)	8
M. Kifle et al. [20]	Manufacturing Industry	Addis Ababa	2014	Cross-sectional	444	98	33.30 (28.92, 37.68)	8
Tanga et al. [26]	Manufacturing Industry	Arba Minch	2018	Cross-sectional	412	100	20.60 (16.70, 24.505)	6
Osman Yiha, Abera Kumie [36]	Manufacturing Industry	Afar	2010	Cross-sectional	810	97.8	78.30 (75.46, 81.14)	7
Moges et al. [37]	Manufacturing Industry	Kombolcha	2013	Cross-sectional	455	100	36.90 (32.46, 41.33)	6
Gebretsadik et al. [2]	Manufacturing Industry	Muger	2017	Cross-sectional	498	99.6	10.40 (7.72, 13.08)	8
Bona et al. [24]	Manufacturing Industry	Hawassa	2018	Cross-sectional	181	100	38.00 (30.93, 45.07)	8
Gebremichael et al. [27]	Manufacturing Industry	Arba Minch	2015	Cross-sectional	433	98	31.40 (27.03, 35.77)	7
Bogale et al. [21]	Sewerage, Waste Management and Remediation Activities	Adiss Ababa	2014	Cross-sectional	876	97.7	43.70 (40.42, 46.99)	6
Gizaw et al. [32]	Sewerage, Waste Management and Remediation Activities	Gondar and Bahir Dar	2014	Cross-sectional	482	100	63.90 (59.61, 68.19)	8
Eskezia et al. [30]	Sewerage, Waste Management and Remediation Activities	Amhara	2016	Cross-sectional	379	96.2	34.30 (29.52, 39.08)	7
Mersha et al. [22]	Construction	Adiss Ababa	2017	Cross-sectional	806	99.6	84.70 (82.22, 87.19)	8
Tadesse and Israel [23]	Construction	Addis Ababa	2016	Cross-sectional	504	92.6	38.30 (34.06, 42.54)	6
Kaweti and Abegaz [25]	Human Health and Social Work Activities	Hawassa	2016	Cross-sectional	496	94.3	28.00 (24.05, 31.95)	7
Abebe et al. [31]	Human Health and Social Work Activities	Amhara	2017	Cross-sectional	193	90.6	18.70 (13.20, 24.20)	7
Bekele et al. [38]	Human Health and Social Work Activities	Bale	2015	Cross-sectional	340	93.9	37.10 (31.97, 42.24)	8
A. Lette et al. [39]	Construction	Jimma	2018	Cross-sectional	355	98.6	41.40 (36.28, 46.52)	6
Adane et al. [28]	Construction	Gondar	2013	Cross-sectional	401	99.5	38.70 (33.93, 43.47)	6
Walle et al. [40]	Human Health and Social Work Activities	Bahir Dar	2013	Cross-sectional	332	100	31.00 (26.03, 35.98)	8
G. Kebede et al. [29]	Human Health and Social Work Activities	Gondar	2011	Cross-sectional	344	95.3	30.80 (25.92, 35.68)	7

### Prevalence of occupational injury in Ethiopia

**Fig. 2:**
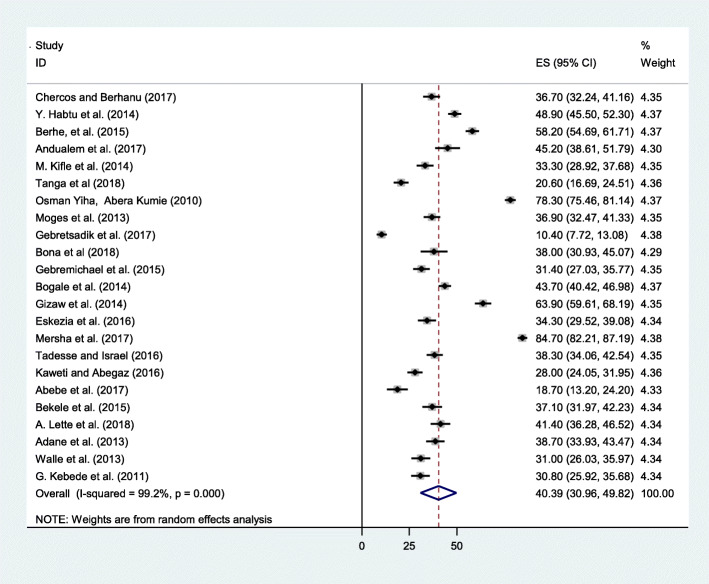
Forest plot of the pooled prevalence of occupational injuries in Ethiopia, 2019

As indicated in the forest plot, the pooled prevalence of occupational injury in Ethiopia was 44.66% (95% CI: 43.83, 45.49) (Fig. 2). However, substantial heterogeneity was revealed across studies (I^2^ = 99.2%, *p* ≤ 0.001). By considering this fact, random effect analysis was conducted.

### Publication bias

**Fig. 3:**
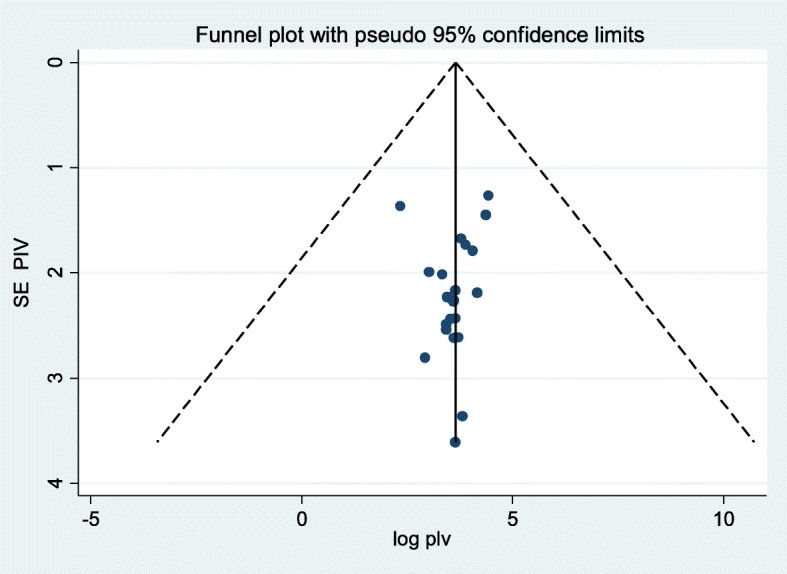
Funnel plots for publication bias of prevalence and associated factors of occupational injuries in Ethiopia, 2019

Since this review had a substantial form of heterogeneity, we assessed publication bias using funnel plot (Fig. 3). As subjectively described below in the funnel plot, the scatter plots of each study are less concentrated near to zero indicating that there is publication bias or systematic heterogeneity across studies. Hence, as a means of confirmatory test; both Begg’s and Egger’s tests were considered. The result of both Begg’s and Egger’s tests indicated that there was no any type of publication bias at *p* value = 0.526 and 0.061 respectively.

### Subgroup analysis

To identify the presence of heterogeneity, subgroup analysis was also conducted by considering region, study year and the site of injury.

Based on the subgroup analysis, the highest prevalence of occupational injury was reported from Addis Ababa city administration (49.5%) followed by Amhara national regional state (36.38%). Moreover, there was also highest injury reported from construction sites (50.8%) followed by sewerage, waste management and remediation sites (47.31%), (Table 2).

**Table 2:** Subgroup analysis of the prevalence of occupational injury in Ethiopia

By Region		Heterogeneity statistic	Number of studies	Prevalence (95% CI)	*P*-values	I-squared (%)	Tau-squared
Addis Ababa		748.16	5	49.82(28.87, 70.76)	< 0.001	99.5	567.46
SNNP		48.53	5	32.28 (24.69,39.86)	< 0.001	91.8	67.67
Amhara		206.05	7	36.38 (25.80, 46.95)	< 0.001	97.1	197.64
Oromo		158.58	3	29.55 (7.78, 51.31)	< 0.001	98.7	364.75
Occupational site							
Manufacturing Industry		1420.70	11	39.81(25.79, 53.84)	< 0.001	99.3	558.01
Sewerage, Waste Management and Remediation Activities		90.62	3	47.31 (31.46,63.17)	< 0.001	97.8	191.75
Construction		590.27	5	50.82 (22.97,78.67)	< 0.001	99.5	802.92
Human Health and Social Work Activities		24.35	6	29.17 (23.82,34.51)	< 0.001	83.6	30.93
study year	< MDGs (< 2015)	663.10	9	45.12 (33.08, 57.13)	< 0.001	98.8	334.30
	≥MDGs (≥2015)	2025.56	14	37.37 (23.72, 51.02)	< 0.001	99.4	673.13

### Meta-regression

Beyond subgroup analysis, meta-regression for the included studies was also conducted to identify factors for heterogeneity. However, there was no statistical significance value from the meta-regression model (Table 3).

**Table 3:** Meta-regression of the included studies to estimate the pooled prevalence of occupational injury and its associated factors in Ethiopia

Variables	Characteristics	Coefficient	*P*-value
Publication year		−0.06	0.76
Sample size		0 .01	0.51
Region	Addis Ababa	3.58	0.77
	Amhara	−10.14	0.346
	Oromia	−7.25	0.64
	SNPs	−13.95	0.23
Site of injury	Manufacturing Industry	−11.08	0.28
	Human Health and Social Work Activities	−21.77	0.07
	Sewerage, Waste Management and Remediation Activities	−3.59	0.79
	Construction	Reference	Reference

### Sensitivity analysis

**Fig. 4:**
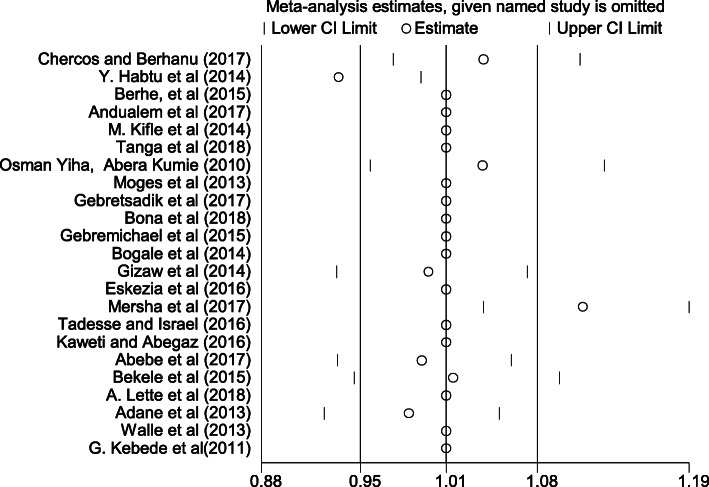
Sensitivity analysis which indicates outlier studies of this systematic review and meta-analysis

Sensitivity analysis was done to identify outlier studies. Previous studies have shown that when data points are outliers, they can be omitted from statistical analysis [41]. In the systematic review, reporting of sensitivity analysis can best be done by influential meta-analysis. If results remain consistent across the different analysis and sensitivity analysis tests, the results may be considered stable. If the findings vary across sensitivity analysis, it is an indicator of careful interpretation of the sensitivity analysis output. Once more, subjectively, we considered the articles are influential if the graph for each corresponding articles outlines either from its corresponding lower confidence interval or upper confidence interval. According to the analysis, Chercos and Berhanu [33], Y. Habtu et al. [9], Osman Yiha, Abera Kumie [36] and Mersha et al. [22] influenced the analysis and dropped for the final model analysis (Fig. 4).

### Associated factors of occupational injury in Ethiopia

Male workers, working more than eight hours per day, respondents who did not use personal protective equipment, lack of supervision, and lack of training about occupational health and safety were found as the factors of occupational injury in Ethiopia.

**Fig. 5:**
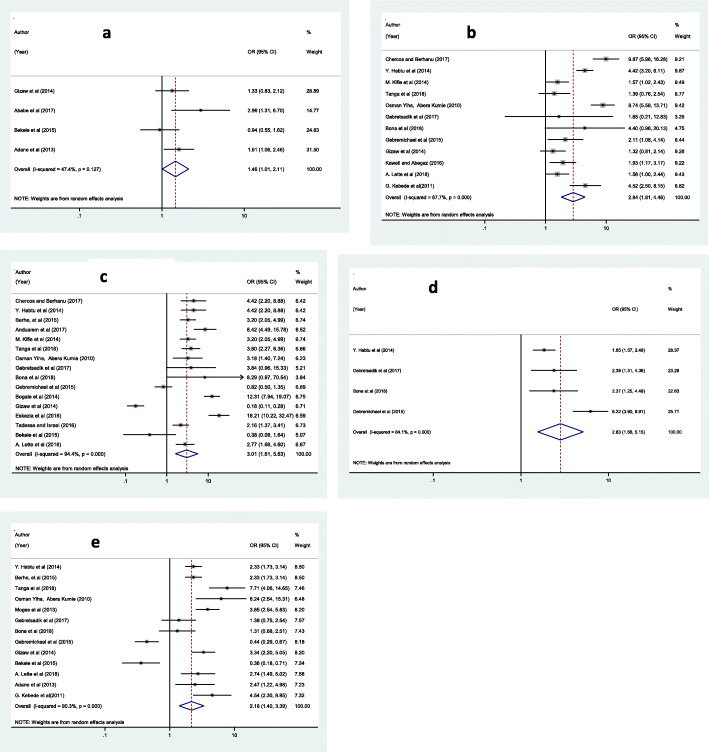
Forest plots which describe: **a)** the association between sex difference in occupational injury; **b)** the association between work duration and occupational injury; **c)** the association between PPE and occupational injury; **d)** the association between workplace Supervision and occupational injury; **e)** the association between occupational health and safety training and occupational injury in Ethiopia

Workers who are males had the odds of 1.46 to experience occupational injury as compared to female workers [OR = 1.46 (95% CI: 1.01, 2.11)] (Fig. 5a). Individuals who had history of work engagement more than eight hours per day had also the odds of 2.84 to experience occupational injury as compared to those who had history of work engagement less than or equal to eight hours per day [OR = 2.84 (95% CI: 1.81, 4.46)] (Fig. 5b). The odds of occupational injury were also 3.01 times higher among individuals who did not use Personal Protective Equipment (PPE) as compared to those individuals who used PPE [OR = 3.01 (95% CI: 1.61, 5.63)] (Fig. 5c). Moreover, the odds of having occupational injury were 2.83 times higher among individuals who had not health and safety supervision at work than among individuals who had supervision [OR = 2.83 (95% CI: 1.58, 5.15)] (Fig. 5d). Once more, the odds of occupational injury were 2.18 times higher among individuals who had no training about occupational health and safety than individuals who had training about occupational health and safety [OR = 2.18 (95% CI: 1.40, 3.39)] (Fig. 5e).

## Discussion

The aim of this systematic review and meta-analysis was to estimate the pooled prevalence of occupational injury and its associated factors in Ethiopia. This finding reported that the pooled prevalence of occupational injury was 40.39% (95% CI: 30.96, 49.82). This finding was in line with the study done in Ghana 40% [42]. This can be attributed to many work-related accidents in developing countries. This is due to the fact that poor working conditions, lack of effective injury prevention systems, low health and safety regulations, low wages for workers and low social benefits [43].

However, our finding was higher than the study done in Norway 31.70% [44], Brazil 5.6% [45], Nigeria 13.5% [46], and Turkey 28.7% [47]. Likewise, the pooled prevalence was lower than the study done in Japan 44.20% [48], India 49.70% [49], Iran 75.4% [50], Egypt 46.2% [51], Colombo 43.7% [52], Zimbabwe 41% [53] Greek 2.4% [54]. This can be possibly justified by that work-related injury occur in low- and middle-income countries with the highest proportion of the world’s population and the highest proportion of workers in risky jobs; whereas high-income countries also account for a significant number of work-related deaths [43]. From the current review, the government has not prioritized occupational safety and health to tackle occupational health problems, and is likely to have a low level of occupational health inspections, recording workplace accidents, and lack of effective surveillance systems.

From the subgroup analysis, the prevalence of occupational injury showed a sluggish decrement after the period of MDGs (37.37%) in comparison with before the end of the MDGs (45.12%). This is a clue for the concerned bodies to effectively implement legislations to minimize occupational injury and related consequences. The highest prevalence of injury was also reported from construction sites (50.8%) followed by sewerage, waste management and remediation sites (47.31%).

From the current review, being male was statically associated with occupational injury. This finding was comparable with the study done from Brazil [55], Japan [48] and Finland [56]. Since there is no a well-established evidence previously about how sex is related with occupational injury, indeed; it warrants further investigation.

The individuals who were engaged to work more than eight hours per day were also at great risk of being injured as compared to those who were engaged to work for less than or equal to eight hours per day. This finding was comparable with the study done from Bangladesh [57], USA [58] Nigeria [59] and USA [60]. This could be explained by that work overload my attribute for various emotional, physical and social consequences of employees; this in-turn may expose for occupational injury [61].

Lack of health and safety supervision at the workplace increases the risk of occupational injury amongst the workers. Compatible findings were also reported from Japan [48] and Brazil [55]. For this, professional supervisors may encourage health in the workplace by advising employees if they are within unsafe condition [62].

Moreover, lack of using Personal Protective Equipment (PPE) is also a risk factor for the occurrence of occupational injury. Consistent findings were also reported among studies done from Norway [44] and India [63] and Japan [48]. As the recommendations are already forwarded from WHO, utilization of PPE will reduce exposure to chemical, radiological, physical, electrical, mechanical and/or other hazards.

Likewise a study reported in Japan [48], from the current review; there was also higher reports of occupational injury among individuals’ who did not have occupational health and safety trainings. This is the fact that training activities are the basic programs which instruct employees to avoid known hazards by properly using and maintaining equipment and materials. It proactively help workers to identify and resolve potential problems that may cause occupational injury [64].

In order to minimize this high burden of occupational injury, the concerned body should give special attention to all the identified factors. The consequences of occupational injury can be felt when their work product is not successful and sustainable. The nature of workers ‘exposure to occupational hazards depends on the type of work being carried out. Limited use of PPE, lack of training for staff and poor lighting in places of work were among the causes of such injury. However, the low educational level of the workers, their age, work shift and occupations (for example, weaving or spinning sections) were factors associated with increased risk of injury. Health and health at work to be implemented and ratified for the protection of employees by legislation, regulatory framework and compliance requirements.

Globally, limited workers’ safety coverage and substantial under-reporting of fatal type of occupational injury, disorganized documentation, and incomplete documentation of all data system forms are the main factors leading to underestimation of injury-related occupational deaths [4, 65–67]. In Ethiopia, there is no a comprehensive national surveillance and reporting system for occupational injury. This suggests that the frequency of work-related accidents, illnesses and even deaths are underestimated. Hence, underreporting of occupational injury reduces our capacity to identify and address occupational health issues. This also impacts both workers and the community that require significant research and treatment. Thus, new programs, models and approaches need to be adapted to identify causes of underreporting of occupational injury in Ethiopia.

### Strengths and limitations

Primarily, this systematic review and meta-analysis used internationally accepted tools for critical appraisal system for quality assessment of individual studies. It included published and unpublished articles. By including data from unpublished studies and gray literature, this meta-analysis and systematic reviews could account for publishing bias due to under-reporting negative results, which contributes to bias in meta-analysis, thus misinforming researchers and policymakers.

## Conclusion

Based on this systematic review and meta-analysis, it is concluded that nearly half of the labor workers in Ethiopia were experienced occupational injury. This issue was more encountered among the labor workers of construction sites and whose working place were at the Addis Ababa city administration respectively. Being male sex, working more than eight hours per day, lack of personal protective equipment, lack of supervision, and lack of training about occupational health and safety had increased odds of occupational injury in Ethiopia. Hence, the concerned body should give special emphasis for all the explored factors in order to minimize occupation related injury, mortality and morbidity in the country.

## Data Availability

The datasets analyzed during the current study are available from the corresponding author upon reasonable request.

## Abbreviations

CI: Confidence Interval
OR: Odds Ratio
PPE: Personal Protective Equipment
SNNP: South Nations Nationalities and Peoples of Ethiopia
